# Clinical and Echocardiographic Characterization of Stage B Heart Failure in Asymptomatic Adults With Cardiovascular Risk Factors

**DOI:** 10.7759/cureus.116788

**Published:** 2026-09-23

**Authors:** Muhammad Ayhan Amir, Abdul Hannan, Muhammad Farhan Shabbir, Eemaz Nathaniel, Azat Iarullin Ilgizovich, Faiqa Haleem, Saba Saeed, Muhammad Usman Arshad, Isra Mohamed Idris Abubakr, Shatha BaniKhalaf

**Affiliations:** 1 Internal Medicine, Shifa College of Medicine, Shifa Tameer-e-Millat University, Islamabad, PAK; 2 Internal Medicine, University Hospitals Dorset NHS Foundation Trust, Dorset, GBR; 3 Internal Medicine, University of South China, Hengyang, CHN; 4 Internal Medicine, Sheffield Teaching Hospitals NHS Foundation Trust, Sheffield, GBR; 5 Interventional Cardiology, Ch. Pervaiz Elahi Institute of Cardiology, Multan, Multan, PAK; 6 Cardiology, Rehman Medical College, Peshawar, PAK; 7 Internal Medicine, City Clinical Hospital No. 12, Kazan, RUS; 8 Internal Medicine, S. M. Kirov Military Medical Academy, Saint Petersburg, RUS; 9 Internal Medicine, Jinnah Sindh Medical University, Karachi, PAK; 10 General Medicine, Ziauddin University, Karachi, PAK; 11 General Medicine, Manchester University NHS Foundation Trust, Manchester, GBR; 12 Medicine, Foundation University Medical College, Rawalpindi, PAK; 13 Medicine, University Hospitals Dorset NHS Foundation Trust, Dorset, GBR; 14 Internal Medicine, Ahfad University for Women, Omdurman, SDN; 15 General Practice, University Hospital Sharjah, Sharjah, ARE; 16 General Practice, Jordan University of Science and Technology, Irbid, JOR

**Keywords:** asymptomatic adults, cardiovascular risk factors, diastolic dysfunction, echocardiography, pre-heart failure, stage b heart failure

## Abstract

Background: Stage B heart failure (pre-HF) is characterized by objective cardiac abnormalities without HF symptoms. This study assessed the prevalence and clinical and echocardiographic characteristics of those who were asymptomatic yet had cardiovascular risk factors and Stage B HF.

Methods: This cross-sectional study included 900 asymptomatic adults with cardiovascular risk factors, consecutively recruited from participating hospitals from October 2025 to July 2026. We performed clinical evaluation and a standardized transthoracic echocardiogram. Stage B/pre-HF was defined as the presence of objective cardiac structural or functional abnormalities in the absence of heart failure symptoms or signs. We evaluated independent factors associated with Stage B/pre-HF using multivariable logistic regression.

Results: In this hospital-based cohort, Stage B/pre-HF was determined in 261 (29.0%) participants. Hypertension, diabetes mellitus, and obesity were more prevalent in the Stage B group, occurring in 154 (59.0%), 118 (45.2%), and 109 (41.8%), respectively. Stage B/pre-HF was characterized descriptively by lower left ventricular ejection fraction and higher left ventricular mass index, left atrial volume index, E/e′ ratio, and tricuspid regurgitation velocity, along with impaired diastolic function. Hypertension was the strongest independent association (OR = 5.930, 95% CI: 3.815-9.217), followed by diabetes (OR = 2.945, 95% CI: 1.913-4.532), age, sex, and BMI. The model showed good fit (Nagelkerke R² = 0.565; Hosmer-Lemeshow p = 0.449).

Conclusion: Stage B/pre-HF was common among asymptomatic adults with cardiovascular risk factors and was characterized by structural and functional cardiac abnormalities. These findings support the potential value of early identification and management of modifiable cardiovascular risk factors in asymptomatic adults at risk for HF.

## Introduction

Heart failure (HF) is a progressive process that starts with cardiovascular risk factors, followed by asymptomatic structural and functional cardiac abnormalities, and culminates in a symptomatic HF stage. Stage B HF (pre-HF) is a clinically relevant asymptomatic period with objective findings indicative of structural or functional cardiac abnormalities but without clinical signs or symptoms of HF [1,2]. Adverse ventricular remodeling and impaired cardiac function are related to common cardiovascular risk factors, such as hypertension, diabetes mellitus, obesity, and dyslipidemia, which can occur before clinical HF [3,4].

Echocardiography provides a non-invasive method for identifying early structural and functional cardiac abnormalities, including ventricular remodeling, chamber enlargement, and impaired diastolic function, in asymptomatic individuals at risk for HF [5,6]. Left ventricular ejection fraction (LVEF), left ventricular mass index (LVMI), left atrial volume index (LAVI), and E/e′ ratio can help characterize subclinical changes in ventricular structure and filling pressure [7]. Clinically, these abnormalities have been detected in asymptomatic patients because Stage B HF has been associated with a higher risk of cardiovascular events and progression to HF symptoms [8,9].

Despite the clinical importance of pre-HF, the clinical and echocardiographic characteristics of Stage B HF among hospital-recruited, asymptomatic adults with common cardiovascular risk factors remain insufficiently characterized. Previous community-based studies have shown that structural, systolic, and diastolic abnormalities are associated with different cardiovascular risk factors, and selected echocardiographic markers have been studied for their prognostic value [10-12]. Therefore, this study aimed to determine the prevalence of Stage B/pre-HF among asymptomatic adults with cardiovascular risk factors. The secondary objectives were to describe the clinical and echocardiographic characteristics of participants with Stage B/pre-HF and to identify clinical factors independently associated with Stage B/pre-HF, using multivariable logistic regression.

## Materials and methods

Research framework

This cross-sectional observational study included adults aged ≥18 years who had at least one cardiovascular risk factor, were asymptomatic for HF, and had no clinical signs of HF. We recruited participants consecutively from Shifa International Hospital, Islamabad, Pakistan, during the study period from October 2025 to July 2026. The study characterized the clinical and echocardiographic features of asymptomatic adults at cardiovascular risk and assessed the presence of Stage B/pre-HF.

Eligibility criteria and participant characteristics

Adults (aged ≥18 years) with at least one cardiovascular risk factor (hypertension, diabetes mellitus, dyslipidemia, obesity, and smoking) were included in the study population. One or more risk factors were sufficient for eligibility, and participants did not need multiple cardiovascular risk factors. Participants who had previously diagnosed HF, current HF symptoms or signs, significant symptomatic cardiovascular disease that required urgent treatment, acute myocardial infarction and acute coronary syndrome, known significant valvular disease or congenital heart disease that could significantly affect cardiac structure or function, poor echocardiographic image quality, or any condition that could affect echocardiographic assessment of the heart were excluded.

Before enrollment, all participants underwent standardized screening for symptoms suggestive of HF. Participants were specifically asked about dyspnea, orthopnea, paroxysmal nocturnal dyspnea, unexplained peripheral edema, unexplained exercise intolerance, and unusual fatigue attributable to cardiac disease. Those with current symptoms or signs of HF were excluded to keep the study population within the asymptomatic/pre-HF spectrum. Participant screening, eligibility assessment, exclusions, and final allocation according to Stage B/pre-HF status are presented in Figure 1.

**Figure 1:**
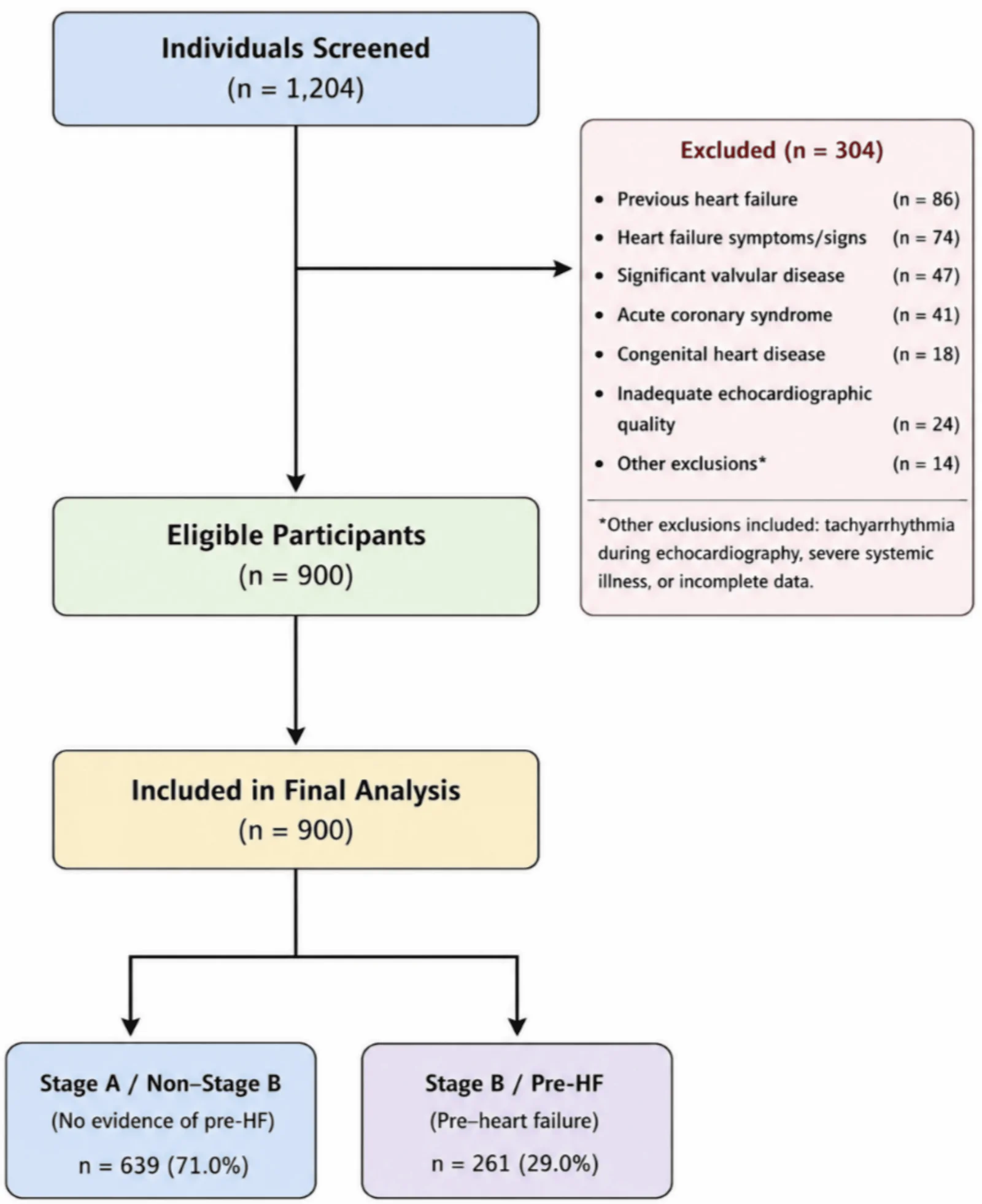
Flow diagram showing screening, eligibility assessment, exclusions, and final allocation of study participants according to Stage B/pre-HF status

Participant recruitment and sampling procedures

We consecutively recruited eligible adults from participating hospitals during the predefined recruitment period. We screened potential participants according to the predefined inclusion and exclusion criteria. We provided study information before obtaining written informed consent, and enrolled all eligible individuals who agreed to participate. This consecutive recruitment approach minimized investigator selection bias.

Determination of the Study Sample

A minimum sample size was determined using the single-proportion formula,

$n = \frac{Z^2 P(1-P)}{d^2}$,

where n was the sample size required, Z was the standard normal deviate at a 95% confidence level (1.96), P was the expected prevalence of Stage B HF/pre-HF, and d was the margin of error desired. Because no reliable prior estimate of the prevalence of Stage B HF/pre-HF in the target population was available, we assumed a conservative prevalence of 50% and a margin of error of 5%, resulting in a minimum sample size of 384 participants. We set an initial recruitment target of 427 participants to account for incomplete clinical information, technically inadequate echocardiographic examinations, and eligibility-related exclusions. During the predefined recruitment period, eligible participants were recruited consecutively from the participating hospitals. Consequently, recruitment exceeded the minimum target, and 900 eligible participants were ultimately included in the final analysis. The larger achieved sample provided greater precision for estimating the prevalence of Stage B/pre-HF and adequate statistical power for the prespecified multivariable logistic regression analysis.

Data collection and clinical evaluation

Demographic and clinical information were collected using informed consent and a data collection form. Demographic measures were age and sex. Risk factors for cardiovascular disease were defined as hypertension, diabetes mellitus, dyslipidaemia, tobacco smoking, and obesity/BMI. Clinical parameters measured were systolic and diastolic blood pressure, heart rate, height, weight, and BMI. We ascertained hypertension, diabetes mellitus, and dyslipidemia as present or absent based on the participant's known medical history or clinical documentation. Smoking status was classified as never smoker, former smoker, or current smoker.

Where feasible, we obtained recent routine laboratory results from available medical or laboratory records rather than requiring additional blood testing solely for research purposes. Laboratory parameters evaluated were HbA1C, LDL cholesterol, serum creatinine, and estimated glomerular filtration rate (eGFR). The study protocol included a pre-defined acceptable time window for existing laboratory results to ensure that laboratory values reasonably reflected the participant's clinical state at the time of assessment.

Echocardiographic assessment

All participants underwent one standardized transthoracic echocardiographic examination performed by two trained cardiologists. All examiners had five years of experience. We performed echocardiographic examinations using a GE Vivid S70N ultrasound system (GE HealthCare, Chicago, USA) equipped with a phased-array cardiac transducer. Participants rested before the examination and were evaluated in the left lateral decubitus position with ECG electrodes in place. Standard parasternal long-axis and short-axis and apical four-chamber, two-chamber, and, where required, three-chamber views were acquired to assess cardiac structure, chamber dimensions, systolic function, and Doppler parameters.

Left ventricular systolic performance was evaluated using the LVEF with the biplane Simpson's method of disks from apical four- and two-chamber views. End-diastolic and end-systolic volumes were obtained by tracing the left ventricular endocardial borders, and LVEF was calculated as $\mathrm{LVEF} = \frac{\mathrm{EDV} - \mathrm{ESV}}{\mathrm{EDV}} \times 100$.

Because all participants had atrial fibrillation, measurements were obtained over five consecutive cardiac cycles and averaged to reduce beat-to-beat variability. This resulted in a continuous percentage LVEF.

Left ventricular structure was assessed by recording interventricular septal thickness, diastolic left ventricular internal diameter, and left ventricular posterior wall thickness. Left ventricular mass was automatically obtained from the echocardiographic system, and the LVMI was calculated as the left ventricular mass divided by the body surface area (BSA) and expressed in g/m². We retained measurements of the underlying structure and the continuous LVMI value for further analysis, not just to classify left ventricular hypertrophy. Left atrial volume was calculated from the standard apical four- and two-chamber views, and LAVI was calculated as mL/m². We used continuous LAVI for analysis.

Pulsed-wave Doppler and tissue Doppler imaging were used to assess diastolic function. Early transmitral filling velocity (E), late transmitral filling velocity (A), and the E/A ratio were determined. Tissue Doppler imaging was used to obtain septal and lateral early diastolic mitral annular velocities (e′), and the E/e′ ratio was calculated according to contemporary echocardiographic recommendations. We obtained all Doppler and tissue Doppler measurements using the same five-cycle averaging approach described above. When technically possible, peak tricuspid regurgitation (TR) velocity was also recorded. Peak TR velocity was measurable in 748 (83.1%) participants. In the remaining 152 (16.9%) participants, a reliable TR Doppler signal could not be obtained for technical reasons, and TR velocity was recorded as missing rather than assigned a value of zero.

We classified diastolic function according to the 2016 recommendations of the American Society of Echocardiography (ASE) and the European Association of Cardiovascular Imaging (EACVI). The prespecified criteria considered septal e′ <7 cm/s, lateral e′ <10 cm/s, average E/e′ >14, LAVI >34 mL/m², and peak TR velocity >2.8 m/s as abnormal. Diastolic dysfunction was considered present when more than half of the available parameters were abnormal, normal diastolic function when fewer than half were abnormal, and indeterminate when exactly half were abnormal. Diastolic function classification was performed separately from the overall Stage B/pre-HF classification [13].

Definition of Stage B heart failure

Stage B HF was characterized by the lack of current heart failure symptoms or signs in the presence of objective evidence of structural or functional cardiac abnormality or evidence of increased cardiac filling pressures. Participants were categorised as Stage B/pre-HF if they had no signs or symptoms of HF at the time of the study but had at least one cardiac abnormality, as determined by echocardiographic evaluation. In this study, qualifying echocardiographic abnormalities included reduced LV systolic function, LV hypertrophy, left atrial enlargement, evidence of increased LV filling pressure, and elevated TR velocity. For Stage B/pre-HF classification, a LAVI threshold of ≥29 mL/m² was prespecified as an independent structural criterion, whereas the higher threshold of >34 mL/m² was used specifically as one component of the ASE/EACVI diastolic function algorithm. These thresholds served different purposes and were therefore not interchangeable. We interpreted individual echocardiographic parameters, including LVEF, LVMI, LAVI, E/e′, and diastolic function, according to contemporary echocardiographic criteria. The operational criteria used to characterize Stage B/pre-HF are summarized in Table 1. We therefore assigned the Stage B/pre-HF stage based on the overall clinical and echocardiographic evaluation, not individual echocardiographic parameters. Participants who had cardiovascular risk factors but no heart failure symptoms and signs and no qualifying structural or functional cardiac abnormality were considered as Stage A/non-Stage B.

**Table 1:** Operational definition of Stage B/pre-HF HF = heart failure; LVEF = left ventricular ejection fraction; LVMI = left ventricular mass index; LAVI = left atrial volume index; TR = tricuspid regurgitation

Criterion	Operational definition
Reduced LV systolic function	LVEF <50%
LV hypertrophy	LVMI >116 g/m² in men or >95 g/m² in women
Left atrial enlargement	LAVI ≥29 mL/m²
Increased LV filling pressure	Average E/e′ ≥15
Elevated TR velocity	Peak TR velocity >2.8 m/s
Overall Stage B/pre-HF classification	Absence of HF symptoms/signs plus at least one qualifying structural or functional cardiac abnormality or evidence of increased LV filling pressure

Data acquisition and quality assurance

After clinical screening and collection of demographic and clinical information, every eligible participant underwent one standardized transthoracic echocardiographic examination. LVEF, LVMI, LAVI, mitral inflow parameters, tissue Doppler measurements, E/e′ ratio, and TR velocity were measurable, and overall diastolic function was obtained from this examination. We did not conduct separate analyses for each echocardiographic parameter, as all measurements were obtained in the same TTE examination.

The echocardiographic evaluation was performed by the two trained cardiologists described above. Both examiners followed the same standardized imaging and measurement protocols. Echocardiographers were blinded to participants’ HbA1c values and other clinical risk-factor data during image acquisition and measurement. All participants underwent standardized imaging views and measurement procedures, and technically inadequate examinations were identified according to predetermined criteria. All continuous measurements were retained in the study database to allow both continuous and categorical analyses. Data were recorded using unique study identification codes and entered into a password-protected database accessible only to authorized research staff.

Analytical and statistical procedures

Statistical analyses were performed using IBM SPSS Statistics, version 26.0 (IBM Corp., Armonk, USA). All continuous variables were summarized as mean ± standard deviation (SD), 95% confidence intervals (CIs), median, interquartile range (IQR), and range, while categorical variables were summarized as frequencies and percentages. We divided participants into two groups: Stage A/non-Stage B and Stage B/pre-HF. Comparisons were made between the groups of categorical variables using Pearson’s chi-square test (with Fisher’s exact test used when necessary), as well as effect sizes, reported as Cramér’s V; continuous clinical and laboratory variables were compared between groups using the Mann-Whitney U test, with effect sizes calculated as r = |Z|/√N. Echocardiographic parameters used in the operational definition of Stage B/pre-HF were summarized descriptively by group and were not subjected to inferential between-group testing. Multivariable binary logistic regression was used to assess the independent clinical factors associated with Stage B/pre-HF status; the dependent variable was Stage B/pre-HF status and the independent variables were age, sex, hypertension, diabetes mellitus, dyslipidemia, smoking status, and BMI. The final model included eight estimated predictor parameters (age, sex, hypertension, diabetes mellitus, dyslipidemia, two indicator parameters for smoking status, and BMI), with 261 Stage B/pre-HF events, corresponding to approximately 32.6 events per parameter. Odds ratios (ORs), 95% CIs, regression coefficients, standard errors, Wald statistics, and p-values were reported. The model was evaluated using the Hosmer-Lemeshow goodness-of-fit test, and Cox and Snell and Nagelkerke pseudo-R² values were reported. Where applicable, the number of participants with which each analysis was conducted was reported, and missing observations were dealt with on an analysis-by-analysis basis. All statistical tests were two-tailed, and a p-value <0.05 was considered statistically significant.

Ethics and regulatory compliance

The appropriate IRB/Ethics Committee of Shifa College of Medicine (#0304-25) approved the study protocol before implementation. The study obtained written informed consent from all participants before enrollment. Participation was voluntary, and participants were informed of the study’s purpose and procedures and their right to decline participation or withdraw at any stage without consequences for their routine medical care. The study included clinical evaluation, examination of available routine laboratory parameters, and a non-invasive transthoracic echocardiogram. No invasive procedures were performed solely for research purposes. The participants’ anonymity was preserved during the study, with coded study identifiers used to represent the information collected rather than identifying information in the analytical dataset. Data were collected and stored securely and were accessible only to authorized research staff.

Study reporting

The study was reported according to the Strengthening the Reporting of Observational Studies in Epidemiology (STROBE) guidelines. The process by which participants were selected, the exclusion criteria, missing data, the clinical and echocardiographic characteristics, and the statistical analyses performed were reported transparently.

## Results

Table 2 shows descriptive characteristics of the 900 study participants. Participants’ age was 56.69 ± 11.50 years; the median age was 57 years, and the range was 18-82 years. Mean BMI was 27.41 ± 4.11 kg/m², indicating that participants were, on average, overweight. Mean systolic and diastolic blood pressure were 121.29 ± 12.47 mmHg and 77.21 ± 8.13 mmHg, respectively, and the mean heart rate was 73.03 ± 8.15 beats/min. The mean HbA1c was 5.85 ± 0.83%, and the mean LDL-C was 118.51 ± 26.56 mg/dL. Renal function was generally preserved, with a mean creatinine level of 0.93 ± 0.19 mg/dL and eGFR of 86.44 ± 13.47 mL/min/1.73 m². Cardiac parameters showed a mean LVEF of 61.07 ± 5.19%, LVMI of 89.54 ± 14.57 g/m², and LAVI of 27.84 ± 6.14 mL/m², while the mean E/e′ ratio was 7.53 ± 1.98. Overall, the findings indicate that the study population consisted predominantly of middle-aged to older adults with mildly elevated BMI, generally normal blood pressure and heart rate, preserved renal function and left ventricular systolic function, and generally unremarkable echocardiographic indices at the population level.

**Table 2:** Descriptive characteristics of study participants (N = 900) M = mean; SD = standard deviation; CI = confidence interval; IQR = interquartile range; BMI = body mass index; BP = blood pressure; LDL-C = low-density lipoprotein cholesterol; eGFR = estimated glomerular filtration rate; LVEF = left ventricular ejection fraction; LVMI = left ventricular mass index; LAVI = left atrial volume index

Variable	M ± SD	95% CI for mean	Median	IQR	Range
Age, years	56.69 ± 11.50	(55.93, 57.44)	57.00	16.00	18–82
BMI, kg/m²	27.41 ± 4.11	(27.14, 27.68)	27.60	5.70	18.50–40.20
Systolic BP, mmHg	121.29 ± 12.47	(120.47, 122.11)	120.50	18.00	90–160
Diastolic BP, mmHg	77.21 ± 8.13	(76.68, 77.74)	77.00	10.00	55–110
Heart rate, bpm	73.03 ± 8.15	(72.50, 73.57)	73.00	12.00	48–98
HbA1c, %	5.85 ± 0.83	(5.80, 5.91)	5.70	0.90	4.40–8.70
LDL-C, mg/dL	118.51 ± 26.56	(116.77, 120.25)	117.00	34.00	45–199
Creatinine, mg/dL	0.93 ± 0.19	(0.92, 0.94)	0.92	0.28	0.45–1.58
eGFR, mL/min/1.73 m²	86.44 ± 13.47	(85.56, 87.32)	87.00	18.00	49–124
LVEF, %	61.07 ± 5.19	(60.73, 61.41)	61.40	6.70	42.00–75.80
LVMI, g/m²	89.54 ± 14.57	(88.59, 90.50)	88.60	20.08	52.70–136.90
LAVI, mL/m²	27.84 ± 6.14	(27.44, 28.24)	27.40	8.38	14.00–49.20
E/e′ ratio	7.53 ± 1.98	(7.40, 7.66)	7.20	2.40	3.70–19.40

Table 3 displays the categorical features of the 900 study subjects. There was a slightly higher proportion of female patients than males (503, 55.9% vs. 397, 44.1%). Among the participants, 249 (27.7%) had hypertension, 204 (22.7%) had diabetes mellitus, and 470 (52.2%) had dyslipidemia, which was the most common cardiometabolic condition identified among the participants. As for smoking status, 538 (59.8%) had never smoked, 215 (23.9%) were former smokers, and 147 (16.3%) were current smokers. Obesity, defined as a BMI ≥30 kg/m², was present in 230 (25.6%) participants, whereas 670 (74.4%) were not obese. Regarding diastolic function, 530 (58.9%) had normal diastolic function, 214 (23.8%) had indeterminate findings, and 156 (17.3%) had diastolic dysfunction. In general, the sample had a high prevalence of cardiometabolic risk factors, including dyslipidemia, hypertension, diabetes mellitus, and obesity, but most individuals had normal diastolic function.

**Table 3:** Categorical characteristics of study participants (N = 900) BMI = body mass index Percentages are calculated using the full sample of 900 participants.

Variable	n	%
Sex		
Male	397	44.1
Female	503	55.9
Hypertension		
No	651	72.3
Yes	249	27.7
Diabetes mellitus		
No	696	77.3
Yes	204	22.7
Dyslipidemia		
No	430	47.8
Yes	470	52.2
Smoking status		
Never	538	59.8
Former	215	23.9
Current	147	16.3
Obesity (BMI ≥30 kg/m²)		
No	670	74.4
Yes	230	25.6
Diastolic function		
Normal	530	58.9
Indeterminate	214	23.8
Diastolic dysfunction	156	17.3

Table 4 presents the prevalence and classification of Stage B/pre-HF among the 900 study participants. Most participants (639, 71.0%) were classified as Stage A/non-Stage B, defined as having cardiovascular risk factors, but not symptoms or signs of HF and no objective structural or functional cardiac abnormalities. In contrast, 261 (29.0%) participants were classified as Stage B/pre-HF, demonstrating objective structural or functional cardiac abnormalities or evidence of increased cardiac filling pressure despite the absence of overt HF symptoms or signs. Overall, a significant proportion of the study population met criteria for Stage B/pre-HF (261, 29.0%).

**Table 4:** Prevalence and classification of Stage B/pre-HF among study participants (N = 900) HF = heart failure Participants classified as Stage A/non-Stage B had cardiovascular risk factor(s) but no HF symptoms/signs and no objective structural or functional cardiac abnormality. Participants classified as Stage B/pre-HF had no HF symptoms/signs but demonstrated objective structural/functional cardiac abnormality or evidence of increased filling pressure.

Heart failure stage	n	%
Stage A/non-Stage B	639	71.0
Stage B/pre-HF	261	29.0
Total	900	100.0

Table 5 summarizes the echocardiographic abnormalities observed among participants classified as Stage B/pre-HF. Diastolic dysfunction was the most frequently observed abnormality, present in 156 (59.8%) participants, followed by LV hypertrophy in 151 (57.9%) and left atrial enlargement in 141 (54.0%) participants. Elevated TR velocity was observed in 106 (40.6%) participants, whereas reduced LVEF was present in 68 (26.1%). Only 3 (1.1%) participants met the prespecified threshold for increased LV filling pressure based on an average E/e′ ratio of ≥15. Because participants could have more than one abnormality, these findings were not mutually exclusive.

**Table 5:** Distribution of echocardiographic abnormalities among participants classified as Stage B/pre-HF (n = 261) HF = heart failure; LVEF = left ventricular ejection fraction; LVMI = left ventricular mass index; LAVI = left atrial volume index Values are presented as frequency (n) and percentage of participants classified as Stage B/pre-HF (n = 261). Participants could meet more than one qualifying criterion; therefore, component frequencies are not mutually exclusive, and percentages do not sum to 100%. All participants classified as Stage B/pre-HF met at least one qualifying structural or functional cardiac abnormality.

Qualifying component	n	%
Abnormal LVEF	68	26.1
Abnormal LVMI	151	57.9
Abnormal LAVI	141	54.0
Diastolic dysfunction	156	59.8
Increased LV filling pressure (average E/e′ ≥15)	3	1.1
Elevated TR velocity (>2.8 m/s)	106	40.6
Had ≥1 qualifying abnormality	261	100.0

Table 6 shows that sex, hypertension, diabetes mellitus, and obesity were significantly associated with Stage B/pre-HF status (p < 0.001). There were more female patients who were classified as Stage B/pre-HF (170, 65.1%) than as Stage A/non-Stage B (333, 52.1%). Hypertension showed the strongest association, with 154 (59.0%) Stage B/pre-HF participants being hypertensive compared with 95 (14.9%) in the Stage A/non-Stage B group (Cramér’s V = 0.448), followed by diabetes mellitus (118, 45.2% vs. 86, 13.5%; V = 0.344) and obesity (109, 41.8% vs. 121, 18.9%; V = 0.237). There were no significant associations between dyslipidemia (p = 0.354) and smoking status (p = 0.079) and Stage B/pre-HF status.

**Table 6:** Association of categorical cardiovascular risk factors with Stage B/pre-HF status (N = 900) HF = heart failure; BMI = body mass index Percentages are calculated within Stage B/pre-HF status. Pearson’s chi-square test was used for all comparisons. Degrees of freedom were 1 for binary variables and 2 for smoking status, which had three categories. No expected cell count was <5; therefore, the assumptions of the Pearson chi-square test were satisfied. Dashes indicate that the statistical values are reported for the variable as a whole. ***p < 0.001

Variable	Stage A/non-Stage B, n (%)	Stage B/pre-HF, n (%)	χ²	p	Cramér’s V
Sex					
Male	306 (47.9)	91 (34.9)	12.75	<0.001^***^	0.119
Female	333 (52.1)	170 (65.1)	—	—	—
Hypertension					
No	544 (85.1)	107 (41.0)	180.39	<0.001^***^	0.448
Yes	95 (14.9)	154 (59.0)	—	—	—
Diabetes mellitus					
No	553 (86.5)	143 (54.8)	106.58	<0.001^***^	0.344
Yes	86 (13.5)	118 (45.2)	—	—	—
Obesity (BMI ≥ 30 kg/m²)					
No	518 (81.1)	152 (58.2)	50.75	<0.001^***^	0.237
Yes	121 (18.9)	109 (41.8)	—	—	—
Dyslipidemia					
No	299 (46.8)	131 (50.2)	0.86	0.354	0.031
Yes	340 (53.2)	130 (49.8)	—	—	—
Smoking status					
Never smoked	397 (62.1)	141 (54.0)	5.07	0.079	0.075
Former smoker	144 (22.5)	71 (27.2)	—	—	—
Current smoker	98 (15.3)	49 (18.8)	—	—	—

Table 7 shows significant differences between the Stage A/non-Stage B and Stage B/pre-HF groups for age, BMI, systolic BP, diastolic BP, HbA1c, and eGFR (all p < 0.001). Systolic and diastolic blood pressure and HbA1c were significantly higher in the Stage B/pre-HF group, while age, BMI, and eGFR also differed significantly between groups. LDL-C levels did not differ significantly between groups (p = 0.392). The strongest associations were with age (r = 0.469) and eGFR (r = 0.432), which showed moderate associations with Stage B/pre-HF status, while systolic BP also showed a moderate association (r = 0.333).

**Table 7:** Comparison of clinical and laboratory characteristics according to Stage B/pre-HF status using the Mann-Whitney U test (N = 900) HF = heart failure; BMI = body mass index; BP = blood pressure; LDL-C = low-density lipoprotein cholesterol; eGFR = estimated glomerular filtration rate; IQR = interquartile range; U = Mann-Whitney U statistic Effect size was calculated as r = |Z|/√N. Dashes indicate values not separately reported for the second row because the Mann-Whitney U, Z, p, and effect-size statistics apply to the overall two-group comparison. ***p < .0001 was considered significant.

Variable	Stage B group	n	Median (Q1–Q3)	Mean rank	Sum of ranks	U	Z	p	Effect size (r)
Age, years	Stage A/non-Stage B	639	53 (46–61)	372.62	238,103.00	33,623.00	−14.07	<0.001***	0.469
	Stage B/pre-HF	261	65 (58–72)	641.18	167,347.00	—	—	—	—
BMI, kg/m²	Stage A/non-Stage B	639	26.9 (23.9–29.3)	409.21	261,484.50	57,004.50	−7.46	<0.001***	0.249
	Stage B/pre-HF	261	29.1 (26.2–31.8)	551.59	143,965.50	—	—	—	—
Systolic BP, mmHg	Stage A/non-Stage B	639	118 (110–126)	395.28	252,581.00	48,101.00	−9.98	<0.001***	0.333
	Stage B/pre-HF	261	128 (118.5–136.5)	585.7	152,869.00	—	—	—	—
Diastolic BP, mmHg	Stage A/non-Stage B	639	76 (71–81)	413.85	264,451.00	59,971.00	−6.62	<0.001***	0.221
	Stage B/pre-HF	261	80 (75–86)	540.23	140,999.00	—	—	—	—
HbA1c, %	Stage A/non-Stage B	639	5.6 (5.2–6.0)	402.69	257,318.00	52,838.00	−8.64	<0.001***	0.288
	Stage B/pre-HF	261	6.1 (5.5–7.0)	567.56	148,132.00	—	—	—	—
LDL-C, mg/dL	Stage A/non-Stage B	639	119 (101–136)	455.24	290,898.00	80,361.00	−0.86	0.392	0.029
	Stage B/pre-HF	261	115 (101–131)	438.9	114,552.00	—	—	—	—
eGFR, mL/min/1.73 m²	Stage A/non-Stage B	639	90 (82–98)	522.23	333,704.50	37,554.50	−12.96	<0.001***	0.432
	Stage B/pre-HF	261	77 (68–86)	274.89	71,745.50	—	—	—	—

Table 8 presents descriptive echocardiographic characteristics by Stage B/pre-HF status. Compared with the Stage A/non-Stage B group, participants classified as Stage B/pre-HF had descriptively lower median LVEF, septal e′, and lateral e′ values, and higher median LVMI, LAVI, A velocity, E/e′ ratio, and TR velocity values. The median diastolic function class was also higher in the Stage B/pre-HF group. These descriptive differences are consistent with the echocardiographic features used to classify Stage B/pre-HF status. Because several of these measures contributed directly or indirectly to the classification algorithm, the observed group differences should not be interpreted as independent associations with Stage B/pre-HF status.

**Table 8:** Descriptive echocardiographic characteristics according to Stage B/pre-HF status (N = 900) HF = heart failure; LVEF = left ventricular ejection fraction; LVMI = left ventricular mass index; LAVI = left atrial volume index; TR = tricuspid regurgitation Values are presented descriptively as median (Q1–Q3). TR velocity was available for 748 participants (516 Stage A/non-Stage B and 232 Stage B/pre-HF). Inferential between-group tests were intentionally not performed because several echocardiographic measures, including LVEF, LVMI, LAVI, E/e′, and TR velocity, contributed directly to the operational definition of Stage B/pre-HF, while septal and lateral e′ contributed to the assessment of diastolic function, which also formed part of the Stage B/pre-HF classification. Consequently, between-group differences in these measures are partly inherent to the classification procedure and should not be interpreted as independent associations with Stage B/pre-HF.

Variable	Stage A/non-Stage B, n	Median (Q1–Q3)	Stage B/pre-HF, n	Median (Q1–Q3)
LVEF, %	639	62.55 (59.83–65.38)	261	57.70 (53.30–61.30)
LVMI, g/m²	639	84.90 (77.35–92.93)	261	100.40 (92.00–110.43)
LAVI, mL/m²	639	25.40 (22.30–28.50)	261	34.35 (30.83–37.28)
E velocity, cm/s	639	72.30 (65.60–80.03)	261	73.85 (67.05–82.58)
A velocity, cm/s	639	66.55 (59.30–74.60)	261	74.60 (66.93–83.10)
E/A ratio	639	1.08 (0.93–1.27)	261	1.00 (0.86–1.14)
Septal e′, cm/s	639	9.40 (8.10–10.70)	261	7.00 (6.00–8.40)
Lateral e′, cm/s	639	12.10 (11.10–13.30)	261	10.00 (8.80–11.08)
E/e′ ratio	639	6.80 (5.90–7.70)	261	8.75 (7.40–10.40)
TR velocity, m/s	516	2.30 (2.06–2.50)	232	2.78 (2.48–2.96)
Diastolic function class	639	1 (1–1)	261	3 (2–3)

Table 9 shows that age, sex, hypertension, diabetes mellitus, and BMI were significantly associated with Stage B/pre-HF status. Each additional year of age was associated with an 8.7% increase in odds of Stage B/pre-HF (OR = 1.087, 95% CI: 1.065-1.108), while male sex was independently associated with higher odds of Stage B/pre-HF (OR = 2.044, 95% CI: 1.368-3.055). The strongest significant association was seen with hypertension (OR = 5.930, 95% CI: 3.815-9.217), followed by diabetes mellitus (OR = 2.945, 95% CI: 1.913-4.532). BMI was also independently associated with Stage B/pre-HF, with an 11.1% increase in odds per 1 kg/m² increase (OR = 1.111, 95% CI: 1.058-1.166). Neither dyslipidemia nor smoking status was a significant independent predictor (p = 0.137 and p = 0.142, respectively). The model showed no evidence of significant lack of fit according to the Hosmer-Lemeshow test (χ² = 7.84, p = 0.449), with a Nagelkerke pseudo-R² of 0.565.

**Table 9:** Multivariable binary logistic regression analysis of factors associated with Stage B/pre-HF status HF = heart failure; B = logistic regression coefficient; SE = standard error; OR = odds ratio; CI = confidence interval; BMI = body mass index Model statistics: −2 log likelihood = 630.364; Cox and Snell R² = 0.396; Nagelkerke R² = 0.565; Hosmer-Lemeshow χ²(8) = 7.84; Hosmer-Lemeshow p= 0.449; AUC = 0.86 (95% CI 0.83–0.89). Exp(B) and OR are the same quantity. The dependent variable was Stage B/pre-HF status. The linearity of the logit assumption for the continuous predictors age and BMI was assessed during model reassessment. No important departure from linearity was identified; therefore, both variables were retained as continuous linear terms in the final logistic regression model. Dashes indicate that the statistical values are reported for the variable as a whole.

Predictor	B	SE	Wald	p	Exp(B)/OR	95% CI for OR
Age, years	0.083	0.010	68.89	<0.001	1.087	(1.065, 1.108)
Male sex vs. female sex	0.715	0.205	12.16	<0.001	2.044	(1.368, 3.055)
Hypertension: yes vs. no	1.780	0.225	62.59	<0.001	5.930	(3.815, 9.217)
Diabetes mellitus: yes vs. no	1.080	0.220	24.10	<0.001	2.945	(1.913, 4.532)
Dyslipidemia: yes vs. no	0.290	0.195	2.21	0.137	1.336	(0.912, 1.959)
Smoking status, overall	—	—	3.90	0.142	—	—
Former smoker vs. never smoker	0.020	0.240	0.01	0.934	1.020	(0.637, 1.633)
Current smoker vs. never smoker	0.480	0.260	3.41	0.065	1.616	(0.971, 2.690)
BMI, kg/m²	0.105	0.025	17.64	<0.001	1.111	(1.058, 1.166)
Constant	−10.800	0.980	121.45	<0.001	<0.001	—

## Discussion

The current study characterizes asymptomatic adults with known cardiovascular risk factors who had clinical and echocardiographic evidence of Stage B HF. Overall, 261 (29.0%) participants met the study criteria for Stage B/pre-HF despite having no clinical signs or symptoms of HF, indicating that a substantial proportion of this hospital-based population with cardiovascular risk factors had detectable subclinical cardiac abnormalities. This is in line with previous studies showing significant prevalence of undiagnosed Stage B HF in people with cardiovascular risk factors without symptoms. A recent study that included ED patients found that 59.8% of high-risk asymptomatic adults had Stage B HF, and a study from North India showed that 52.4% of those who had echocardiographic assessment had Stage B HF even if they were not symptomatic for HF [12,14]. The lower prevalence observed in our cohort may reflect differences in the characteristics of the enrolled populations, clinical settings, referral patterns, and operational definitions used to classify Stage B/pre-HF. The ED study included high-risk adults presenting to an emergency department. In contrast, our study included asymptomatic adults with cardiovascular risk factors and excluded those with symptoms or signs of HF and clinically significant cardiovascular disease. Differences in echocardiographic assessment, including the thresholds and types of abnormalities used to define Stage B/pre-HF, may also contribute to the variation in prevalence estimates across studies. Nevertheless, the identification of Stage B HF across different clinical settings supports the concept of HF as a continuum in which structural and functional cardiac abnormalities can precede the development of overt clinical disease [12-15].

Participants with Stage B/pre-HF were significantly more likely to have hypertension, diabetes mellitus, and obesity than those without pre-HF. Hypertension had the highest association with Stage B, followed by diabetes and obesity. Hypertension continued to be the strongest independent association in multivariable analysis, as the odds of Stage B/pre-HF were almost six-fold greater (OR = 5.930, 95% CI: 3.815-9.217). Diabetes mellitus was independently associated with Stage B/pre-HF (OR = 2.945, 95% CI: 1.913-4.532), and advancing age and BMI were each associated with a step-up in the odds of Stage B/pre-HF. These increased prevalence rates are similar to those reported in other studies relating risk factor burden to subclinical cardiac abnormalities. The Echo-SOL (Echocardiographic Study of Latinos) showed a progressive rise in the prevalence and incidence of pre-HF across the different levels of HF risk factors and age groups; a study of asymptomatic adults with type 2 diabetes revealed that age, systolic blood pressure, and BMI were important factors determining markers of Stage B HF [16,17]. The strong association observed for hypertension in the present study is also consistent with evidence that chronic hypertension contributes to structural and functional cardiac remodeling, including left ventricular hypertrophy and subclinical ventricular dysfunction, which may precede overt cardiovascular disease [18]. The strong association observed in our cohort may partly reflect hospital-based recruitment of adults with known cardiovascular risk factors; however, this cannot be determined from the cross-sectional design. Dyslipidemia and smoking, however, did not have an independent association with Stage B/pre-HF after adjusting for other factors. Thus, the role of individual risk factors may differ across populations and by cardiac phenotype. Overall, these results highlight the significance of recognition and management of modifiable cardiometabolic risk factors in the absence of any symptoms of HF.

The echocardiographic profile of participants classified as Stage B/pre-HF showed differences in ventricular structure, systolic function, and diastolic indices. Participants with Stage B/pre-HF had lower median LVEF and higher median LVMI and LAVI than those classified as Stage A/non-Stage B. They also showed lower septal and lateral e′ velocities and a higher E/e′ ratio, while A velocity and E/A ratio also differed descriptively between the groups. The results are broadly consistent with previous studies showing higher LV mass and left atrial volume, lower e′ velocities, and higher E/e′ among individuals with preclinical or Stage B HF, with diastolic abnormalities representing an important manifestation of subclinical cardiac disease [14,19]. However, these echocardiographic differences should be interpreted cautiously because several of the parameters, including LVEF, LVMI, LAVI, E/e′, and TR velocity, contributed directly to the operational definition of Stage B/pre-HF. Therefore, the between-group findings presented in Table 8 are descriptive rather than independent evidence of a distinct echocardiographic phenotype. Nevertheless, the findings support the clinical value of echocardiographic assessment for identifying structural and functional cardiac abnormalities among asymptomatic individuals at risk for HF [11].

Age was also associated with Stage B/pre-HF, with a moderate between-group effect size and an independent association in the regression model. This result is consistent with earlier observations that the burden of pre-HF and asymptomatic cardiac abnormalities rises with age and the burden of cardiovascular risk factors [20,21]. The Copenhagen Heart Failure Risk Study reported a substantial burden of Stage B and C HF in an elderly population with cardiovascular risk factors, with 37.5% of participants classified as Stage B and 18.25% as Stage C [20]. In addition, longitudinal community-based data have demonstrated that individuals without preclinical HF can develop Stage A or Stage B HF over time, supporting the concept of pre-HF as an intermediate stage in the progression of cardiac disease [19,20]. In the present study, age remained independently associated with Stage B/pre-HF after adjustment for other cardiometabolic factors, suggesting that age-related cardiac remodeling may contribute to the development of subclinical abnormalities beyond the effect of individual risk factors alone. At the same time, the attenuation or absence of some associations after multivariable adjustment indicates that other coexisting risk factors may influence the relationship between individual cardiovascular risk factors and pre-HF. Rather, age, adiposity, hypertension, and diabetes may contribute to different aspects of ventricular remodeling and impaired diastolic function. This interpretation is consistent with previous evidence indicating that the clinical predictors of asymptomatic echocardiographic abnormalities may vary according to the specific cardiac phenotype and the combination of risk factors considered [10,21].

In general, the results indicate that changes in the heart can be identified even when the symptoms of HF are absent, and that some traditional cardiovascular risk factors, especially hypertension, diabetes, excess body weight, and older age, are linked to cardiac changes. The final model showed a Nagelkerke pseudo-R² of 0.565 and no significant lack of fit on the Hosmer-Lemeshow test (p = 0.449). These findings highlight the potential value of recognizing structural and functional cardiac abnormalities among high-risk asymptomatic individuals; however, whether such identification improves clinical outcomes or prevents progression to symptomatic HF requires evaluation in prospective longitudinal studies [21]. Taken together with previous community- and hospital-based studies, our findings suggest that the recognition of pre-HF should not rely solely on the presence of symptoms, as clinically silent structural and functional abnormalities may already be detectable in individuals with conventional cardiovascular risk factors. However, prospective studies are needed to establish whether the abnormalities identified in this cohort translate into subsequent symptomatic HF or cardiovascular events.

Clinical implications

The findings suggest that clinicians should remain vigilant for potential subclinical cardiac abnormalities in asymptomatic adults with hypertension, diabetes, obesity, and increasing age. Echocardiographic assessment can identify structural and functional abnormalities that may not be apparent from symptoms alone. However, the present study does not establish that echocardiographic screening or early identification improves clinical outcomes or prevents progression to symptomatic HF. Prospective studies are therefore needed to determine the clinical utility of screening and whether identification of Stage B/pre-HF can inform interventions that improve subsequent outcomes.

Study limitations

Several limitations should be considered when interpreting the findings. First, the cross-sectional design prevents determination of causal or temporal relationships between cardiovascular risk factors and Stage B/pre-HF. It does not allow for the assessment of whether the identified abnormalities subsequently progressed to symptomatic heart failure or cardiovascular events. Second, participants were consecutively recruited from hospital settings and therefore may differ from individuals in the general community, introducing potential hospital-based selection bias and limiting the external validity and generalizability of the findings to broader populations. Third, several echocardiographic parameters, including LVEF, LVMI, LAVI, E/e′, and TR velocity, contributed directly to the operational definition of Stage B/pre-HF. Consequently, the echocardiographic differences presented between groups should be interpreted as descriptive rather than independent evidence of a distinct phenotype. Fourth, reliable TR velocity measurements were unavailable in 152 (16.9%) participants because an adequate Doppler signal could not be obtained, which may have resulted in incomplete assessment of this criterion and potential misclassification in some participants. Fifth, the use of predefined echocardiographic thresholds may have resulted in threshold-related misclassification, particularly among participants whose measurements were close to the diagnostic cutoffs. Sixth, cardiac biomarkers such as brain natriuretic peptide (BNP), N-terminal proBNP (NT-proBNP), and cardiac troponin were not systematically assessed, and advanced echocardiographic measures such as global longitudinal strain were unavailable. Consequently, subtle myocardial dysfunction or biomarker-defined abnormalities may not have been captured. Seventh, inter- and intra-observer reproducibility of echocardiographic measurements was not formally quantified, which may have introduced measurement variability. Finally, the multivariable regression model did not include potentially relevant factors such as duration and treatment of hypertension or diabetes, physical activity, and medication use; therefore, residual confounding cannot be excluded.

Future prospective, multicenter studies involving community-based populations and longitudinal follow-up are warranted to validate the prevalence and clinical significance of Stage B/pre-HF, incorporate cardiac biomarkers and advanced echocardiographic measures, and determine whether the abnormalities identified in asymptomatic individuals predict progression to symptomatic heart failure or subsequent cardiovascular events.

## Conclusions

In conclusion, Stage B/pre-heart failure was identified in 261 (29.0%) asymptomatic adults with cardiovascular risk factors. Stage B/pre-HF was independently associated with higher BMI, diabetes mellitus, male sex, older age, and particularly hypertension. These findings support the importance of recognition and management of cardiovascular risk factors among asymptomatic adults at risk for HF. However, given the cross-sectional design, the findings do not establish causal relationships, demonstrate a benefit of screening or treatment, or determine progression to symptomatic HF. Prospective longitudinal studies are needed to determine whether the identified cardiac abnormalities predict subsequent symptomatic HF or cardiovascular events and to evaluate the potential impact of targeted risk-factor management.

## References

[1] Heidenreich PA, Bozkurt B, Aguilar D (2022). 2022 AHA/ACC/HFSA Guideline for the Management of Heart Failure: a report of the American College of Cardiology/American Heart Association Joint Committee on Clinical Practice Guidelines. J Am Coll Cardiol.

[2] Aoyanagi H, Nochioka K, Sakata Y (2020). Temporal changes in left ventricular ejection fraction and their prognostic impacts in patients with Stage B heart failure. Int J Cardiol.

[3] La Carrubba S, Todaro MC, Zito C (2013). Asymptomatic left ventricular dysfunction and metabolic syndrome: results from an Italian multicenter study. J Cardiovasc Echogr.

[4] Verdecchia P (2004). OR 61: Asymptomatic left ventricular dysfunction: a potent predictor of cardiac complications in hypertension. Am J Hypertens.

[5] Cowley AC, Dattani A, Yeo JL (2024). Prevalence of stage B heart failure in healthy adults and people with type 2 diabetes. Eur Heart J.

[6] Kosmala W, Marwick TH (2020). Asymptomatic left ventricular diastolic dysfunction: predicting progression to symptomatic heart failure. JACC Cardiovasc Imaging.

[7] Haji K, Huynh Q, Wong C, Stewart S, Carrington M, Marwick TH (2022). Improving the characterization of Stage A and B heart failure by adding global longitudinal strain. JACC Cardiovasc Imaging.

[8] Echouffo-Tcheugui JB, Erqou S, Butler J, Yancy CW, Fonarow GC (2016). Assessing the risk of progression from asymptomatic left ventricular dysfunction to overt heart failure: a systematic overview and meta-analysis. JACC Heart Fail.

[9] Odutayo A, Bhatt DL, Sridhar VS (2026). Association between the 2022 AHA/ACC/HFSA heart failure staging and cardiovascular and kidney outcomes in patients with diabetes and kidney disease: a post hoc analysis of the SCORED randomized controlled trial. Circ Heart Fail.

[10] Coller JM, Gong FF, McGrady M (2022). Risk factors for asymptomatic echocardiographic abnormalities that predict symptomatic heart failure. ESC Heart Fail.

[11] Yang H, Negishi K, Wang Y, Nolan M, Saito M, Marwick TH (2016). Echocardiographic screening for non-ischaemic stage B heart failure in the community. Eur J Heart Fail.

[12] Gottlieb M, Schraft E, O'Brien J, Patel D, Peksa GD (2024). Prevalence of undiagnosed stage B heart failure among emergency department patients. Am J Emerg Med.

[13] Nagueh SF, Smiseth OA, Appleton CP (2016). Recommendations for the evaluation of left ventricular diastolic function by echocardiography: an update from the American Society of Echocardiography and the European Association of Cardiovascular Imaging. Eur Heart J Cardiovasc Imaging.

[14] Gupta R, Das T, Suchak MK (2025). Prevalence of stage B heart failure in patients undergoing echocardiography during regular clinical practice. Indian Heart J.

[15] Young KA, Scott CG, Rodeheffer RJ, Chen HH (2021). Progression of preclinical heart failure: a description of Stage A and B heart failure in a community population. Circ Cardiovasc Qual Outcomes.

[16] Kuno T, Vasquez N, April-Sanders AK (2023). Pre-heart failure longitudinal change in a Hispanic/Latino population-based study: insights from the echocardiographic study of Latinos. JACC Heart Fail.

[17] Gulsin GS, Brady E, Marsh AM (2021). Clinical associations with stage B heart failure in adults with type 2 diabetes. Ther Adv Endocrinol Metab.

[18] Tadic M, Cuspidi C, Marwick TH (2022). Phenotyping the hypertensive heart. Eur Heart J.

[19] Young KA, Scott CG, Rodeheffer RJ, Chen HH (2022). Incidence of preclinical heart failure in a community population. J Am Heart Assoc.

[20] Gaborit FS, Kistorp C, Kümler T (2019). Prevalence of early stages of heart failure in an elderly risk population: the Copenhagen Heart Failure Risk Study. Open Heart.

[21] Yang H, Negishi K, Otahal P, Marwick TH (2015). Clinical prediction of incident heart failure risk: a systematic review and meta-analysis. Open Heart.

